# Effects of Dietary Nitrate Supplementation on Performance and Muscle Oxygenation during Resistance Exercise in Men

**DOI:** 10.3390/nu14183703

**Published:** 2022-09-08

**Authors:** Rachel Tan, Adam Pennell, Katherine M. Price, Sean T. Karl, Noelle G. Seekamp-Hicks, Keonabelle K. Paniagua, Grant D. Weiderman, Joanna P. Powell, Luka K. Sharabidze, Isabella G. Lincoln, Justin M. Kim, Madeleine F. Espinoza, Maya A. Hammer, Richie P. Goulding, Stephen J. Bailey

**Affiliations:** 1 Department of Sports Medicine, Pepperdine University, Malibu, CA 90263, USA; 2 Laboratory for Myology, Faculty of Behavioural and Movement Sciences, Amsterdam Movement Sciences, Vrije Universiteit Amsterdam, 1081 HZ Amsterdam, The Netherlands; 3 School of Sport, Exercise and Health Sciences, Loughborough University, Loughborough LE11 3TU, UK

**Keywords:** nitric oxide, weightlifting, beetroot, neuromuscular

## Abstract

The purpose of the current study was to assess the effects of acute and short-term nitrate (NO_3_^−^)-rich beetroot juice (BR) supplementation on performance outcomes and muscle oxygenation during bench press and back squat exercise. Fourteen recreationally active males were assigned in a randomized, double-blind, crossover design to supplement for 4 days in two conditions: (1) NO_3_^−^-depleted beetroot juice (PL; 0.10 mmol NO_3_^−^ per day) and (2) BR (11.8 mmol NO_3_^−^ per day). On days 1 and 4 of the supplementation periods, participants completed 2 sets of 2 × 70%1RM interspersed by 2 min of recovery, followed by one set of repetitions-to-failure (RTF) at 60%1RM for the determination of muscular power, velocity, and endurance. Quadriceps and pectoralis major tissue saturation index (TSI) were measured throughout exercise. Plasma [NO_3_^−^] and nitrite ([NO_2_^−^]) were higher after 1 and 4 days of supplementation with BR compared to PL (*p* < 0.05). Quadriceps and pectoralis major TSI were not different between conditions (*p* > 0.05). The number of RTF in bench press was 5% greater after acute BR ingestion compared to PL (PL: 23 ± 4 vs. BR: 24 ± 5, *p* < 0.05). There were no differences between BR and PL for RTF for back squat or power and velocity for back squat or bench press (*p* > 0.05). These data improve understanding on the ergogenic potential of BR supplementation during resistance exercise.

## 1. Introduction

Nitric oxide (NO) is a ubiquitous signalling molecule that regulates cardiovascular, metabolic, and contractile processes, and as such, manipulation of NO bioavailability has potential implications for exercise performance [1]. In addition to the nitric oxide synthase enzymes, NO can be derived through the nitrate (NO_3_^−^)–nitrite (NO_2_^−^)–NO pathway, which recycles NO oxidation products (NO_3_^−^ and NO_2_^−^) into NO [2]. As a result, there has been great interest in dietary NO_3_^−^ supplementation as a nutritional intervention to enhance NO synthesis and signalling. Briefly, NO_3_^−^ enters the systemic circulation after ingestion and is actively transported into the salivary glands for subsequent excretion into the oral cavity wherein commensal bacteria reduce NO_3_^−^ into NO_2_^−^ [3]. Swallowed NO_2_^−^ re-enters the systemic circulation, and is converted into NO by various enzymatic reactions in tissues [4], particularly in hypoxic [5] and acidic environments [6]. Recent data also indicate that NO_3_^−^ is rapidly stored in human skeletal muscle after ingestion [7,8] presumably to serve as a source of NO_3_^−^ for NO synthesis in response to alterations in local metabolic demand [9]. Indeed, the increase in muscle [NO_3_^−^] attained after NO_3_^−^ supplementation declines during high-intensity exercise, potentially as a result of xanthine-oxidase-mediated reduction to NO_2_^−^ and then NO [9]. Muscle and plasma NO_3_^−^ and NO_2_^−^ attain peak concentrations ~2 to 3 h following NO_3_^−^ ingestion [7,10], which is synchronous with the time window when physiological and ergogenic effects are most likely to be observed [10,11,12,13].

Mechanistically, the ergogenic effects of NO_3_^−^ supplementation are attributed to some combination of reduced high-energy phosphate cost of force production, lowered exercise-induced metabolic perturbation [14], and improved skeletal muscle calcium handling [14,15]. There is also evidence from animal models to suggest augmented physiological responses in fast-twitch (type II) muscle fibres related to calcium handling [16] or the matching of oxygen (O_2_) supply to demand [17,18] after NO_3_^−^ supplementation, although effects on slow-twitch (type I) muscle fibres have been recently reported [19]. However, in humans, NO_3_^−^ supplementation has been reported to enhance performance during exercise protocols designed to recruit a greater proportion of type II fibres (e.g., faster pedalling cadences [20] and high angular knee velocities [21], which supports the postulate that NO_3_^−^ supplementation may be more ergogenic in exercise settings wherein a greater recruitment of type II fibres occurs [22]. Together, these observations imply that NO_3_^−^ ingestion has the potential to augment explosive, powerful, and high-speed muscle contractions since these movements require a greater relative proportion of type II fibre activation [23]. In support of this postulate, a recent meta-analysis found that NO_3_^−^ supplementation increased muscular power output by 5% [24], although it should be recognized that there are also studies reporting NO_3_^−^ ingestion to be ineffective at enhancing power output [25,26,27,28]. To date, the potential for dietary NO_3_^−^ supplementation to impact power has been examined primarily in single leg knee extension [11,21,26,29,30] and cycling exercise [31,32,33,34,35]. As such, it is unclear whether other exercise modalities requiring high-power and high-velocity contractions, such as resistance exercise (i.e., weightlifting), can benefit from NO_3_^−^ supplementation. Furthermore, no studies have directly examined how acute and multiday NO_3_^−^ supplementation impact resistance exercise performance. This is important since it is equivocal whether multiday NO_3_^−^ loading elicits additional enhancements to running and cycling exercise performance compared to acute doses [36,37], and multiday NO_3_^−^ loading has been suggested to be less effective that acute NO_3_^−^ ingestion for improving muscular power [24]. Further work is, therefore, required to elucidate the impact of dosing regimen on resistance exercise performance. 

Compared to running and cycling, relatively few studies have investigated the impact of dietary NO_3_^−^ supplementation on resistance exercise performance (e.g., muscle power output), and studies conducted to date have yielded conflicting results [38,39,40,41,42,43,44]. For example, in lower-body resistance exercise using back squats, an acute moderate NO_3_^−^ dose (~6.4–13.0 mmol or 400–800 mg of nitrate) has been reported to improve [40,43] and have no effect on power output during back squats [40,42]. In upper-body resistance exercise, there has been one study published to date examining the influence of NO_3_^−^ supplementation on power output during bench press, with this study reporting that power and velocity were improved by ~19% and ~7%, respectively [44]. Given that upper-body musculature may comprise a greater proportion of type II fibres [45,46], and that NO_3_^−^ has been suggested to elicit greater physiological responses in type II fibers [22], NO_3_^−^ supplementation may have greater ergogenic potential during upper-body compared to lower-body resistance exercises. The limited number of studies evaluating the ergogenic potential of NO_3_^−^ supplementation on resistance exercise, particularly lower-body resistance exercise, underscores the need for further research on this topic.

In addition to effects on muscle power output, there are equivocal results pertaining to the effect of NO_3_^−^ supplementation on the number of RTF during resistance exercise. For example, RTF has been reported to increase during back squat exercise following NO_3_^−^ ingestion in some [39,40,42] but not all [38] studies. Similarly, dietary NO_3_^−^ supplementation appears to increase RTF in free-weight bench press [44], but not when Smith-machine bench press has been assessed [41,42]. Therefore, further research is required to assess the effects of NO_3_^−^ supplementation on RTF during upper-body and lower-body resistance exercise. In addition, it is unclear whether dietary NO_3_^−^ supplementation impacts physiological processes, such as muscle oxygenation, during recovery periods interspersed throughout resistance exercise protocols, and whether these effects contribute to altered performance.

The purpose of this study was to assess the effects of acute and short-term, multiday dietary NO_3_^−^ supplementation on plasma [NO_3_^−^] and [NO_2_^−^], skeletal muscle oxygenation, and neuromuscular function and muscle endurance performance, during back squat and bench press. We hypothesized that dietary NO_3_^−^ supplementation would enhance physiological and performance variables during back squat and bench press exercise, with greater improvements during the bench press.

## 2. Materials and Methods

### 2.1. Participants 

Fourteen healthy recreationally active men (mean ± SD: age 22 ± 5 years, body mass 84 ± 17 kg, height 1.80 ± 0.06 m) volunteered to participate in this study following a power calculation based on a published report [44] using a power of 0.95 and alpha of 0.05. Recreationally active was defined as individuals who performed resistance exercise at least twice a week and maintained their normal training regimens throughout the experiment. Participants completed a screening and a physical activity readiness questionnaire. The participant exclusion criteria were individuals with contraindications to exercise, cardiometabolic disease, on ergogenic supplementation, females, and smokers. Females were excluded given that sex-differences in the physiological responses to nitrate ingestion may exist [47]. The experimental protocols, risks, and benefits of participating were explained prior to participants providing written informed consent. This study was approved by the Institutional Research Ethics Committee and in accordance with the Declaration of Helsinki, with the exception of registration to a database. 

### 2.2. Experimental Overview

**Figure 1:**
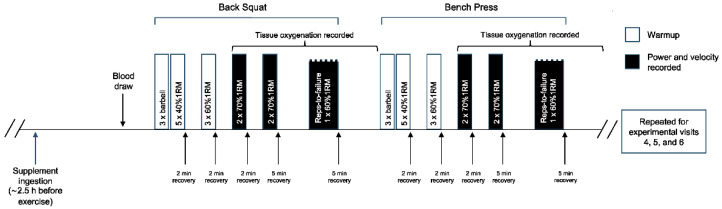
A schematic of the exercise protocol performed on visit 3, 4, 5, and 6 (i.e., experimental visits). 1RM = one repetition maximum.

Participants reported to the laboratory on a total of six occasions over a 3 to 4 wk period. During visit 1, participants underwent standardized one-repetition max (1RM) testing procedures for the determination of the resistance to be applied in subsequent visits. During visit 2, participants performed a protocol and coaching technique familiarization to ensure correct lifting technique. Subsequently, in a double-blind, randomized, crossover design, participants were assigned to two experimental conditions to receive NO_3_^−^-depleted beetroot juice (PL) and NO_3_^−^-rich beetroot juice (BR) for 4 days with a washout period of at least 5 days separating the two supplementation periods. On days 1 and day 4 of each supplementation period, participants reported to the laboratory to perform the experimental protocol (Figure 1).

All tests were performed at the same time of day (±2 h). Prior to the first visit, participants were instructed to avoid antibacterial mouthwash for the duration of the study given that mouthwash has been evidenced to interfere with oral NO_3_^−^ metabolism in humans [48]. Additionally, participants were instructed to refrain from strenuous exercise and alcohol 24 h prior, NO_3_^−^-rich foods (i.e., beetroot, celery, lettuce, radish, spinach, etc.) 48 h prior, and caffeine 12 h prior to each experimental visit. Participants were instructed to record their diet during the 24 h preceding their first experimental visit (visit 3) and to repeat this diet 24 h prior to all subsequent visits.

### 2.3. Exercise Protocols

Participants performed a warm-up in preparation for 1RM testing as previously described [44]. Briefly, participants completed 5 back squat repetitions at 50% of their perceived 1RM, followed by 3 repetitions at 70% of their perceived 1RM with each set interspersed by 2 min of recovery. Subsequently, weight was increased in stepwise increments (0.2 to 9 kg) until the participant’s maximum was successfully lifted within 3 to 5 attempts, with each attempt interspersed by 3 min of recovery. After 3 min of recovery, the same process was repeated to determine the 1RM for the bench press exercise. All participants were required to use standardized procedures for back squat (i.e., medium grip, parallel depth, neutral stance, lower-body extension to original standing position) and bench press (i.e., medium grip, bar to chest, full extension of the arms) throughout the entire duration of the study and were provided coaching techniques. During visit 2, participants performed an explosive lift using the barbell only (20 kg) for a total of 3 repetitions, to ensure correct lifting technique and subsequently completed a familiarization to the exercise protocol for the determination of muscular power and velocity. 

On days 1 and day 4 of each supplementation period (i.e., visit 3, 4, 5, 6), participants reported to the laboratory to perform the experimental protocol to determine muscular power, velocity, and endurance as familiarized during visit 2. A resting venous blood sample was obtained prior to exercise. The movement tempo of individual movement phases during resistance exercise were controlled for using an eccentric–pause–concentric–pause tempo of 1-0-1-2 to emphasize explosive movements and to standardize the lifting cadence across participants [49]. During these visits, participants performed a warm-up prior to the experimental protocol, as previously described [44], consisting of 3 repetitions with the barbell only, followed by 5 repetitions at 40%1RM, followed by 3 repetitions at 60%1RM, with each set interspersed by 2 min of recovery. Following this, a linear position transducer (GymAware, Kinetic Performance Technology, Mitchell, Australia) was attached to the barbell to assess power and velocity of movement. Power and velocity were determined in a protocol consisting of 2 sets × 2 repetitions at 70%1RM with each set interspersed by 2 min of recovery. Following a 5 min recovery period, participants performed 1 set × repetition-to-failure (RTF) at 60%1RM to determine muscular endurance. After a 5 min recovery, the same procedures were repeated in bench press exercise. Participants were instructed to lift the weight as fast as possible during the concentric phase, and encouragement and technical feedback were given to participants during all sets. To optimize the preservation of exercise intensity, smaller muscle groups and fatigue-inducing exercises were performed later within the exercise protocol (e.g., lower body before upper body; unweighted low-rep ballistic exercises before loaded lifts). 

### 2.4. Supplementation Procedures

Participants were randomly assigned to two 4-day supplementation periods in which they consumed 2 × 70 mL doses per day of either concentrated NO_3_^−^-rich beetroot juice (BR: ~5.9 mmol of NO_3_^−^ per 70 mL, Beet it; James White Drinks, Ipswich, UK) or a NO_3_^−^-depleted placebo (PL: 0.05 mmol of NO_3_^−^ per 70 mL, Beet it; James White Drinks, Ipswich, UK) separated by a minimum of a 5-day washout period. On experimental days, which occurred on days 1 and 4 of each supplementation period, participants consumed 2 × 70 mL of their allocated beverage 2.5 h before exercise given that peak plasma [NO_2_^−^] occurs ~2 to 3 h following NO_3_^−^ ingestion [10]. On days 2 and 3 of each supplementation period, participants consumed one 70 mL beverage in the morning and one in the evening. This 4-day protocol was chosen to simulate a potential supplementation regimen an athlete would adopt prior to competition and to provide insight to acute compared to short-term NO_3_^−^ supplementation periods.

### 2.5. Measurements

#### 2.5.1. Blood Analysis 

A resting venous blood sample was obtained from an antecubital forearm vein by a trained member of the research team upon arrival to the laboratory for the assessment of plasma [NO_3_^−^] and [NO_2_^−^]. Samples were drawn into 6 mL lithium heparin tubes (Vacutainer, Becton-Dickinson, NJ, USA) and centrifuged at 3100× *g* at 4 °C for 10 min within 2 min of collection. Plasma was extracted and stored in a −80 °C freezer for the analysis of [NO_3_^−^] and [NO_2_^−^] using gas phase chemiluminescence. All glassware, utensils, and surfaces were rinsed with deionized water to remove NO prior to analysis. Plasma samples were thawed then deproteinized using ice-cold ethanol precipitation prior to [NO_2_^−^] analysis. Specifically, samples were centrifuged at 14,000× *g* for 10 min, and 200 μL of the supernatant was treated with 400 μL of ice-cold ethanol. Samples were then incubated on ice for 30 min, and subsequently centrifuged at 14,000× *g* for 10 min. The [NO_2_^−^] of deproteinized plasma was determined by its reduction to NO using glacial acetic acid and aqueous sodium iodide and calibrated using sodium NO_2_^−^ standards. Following this, the deproteinized plasma samples were diluted prior to [NO_3_^−^] analysis such that 100 μL of the supernatant was added to 400 μL of deionized water. The [NO_3_^−^] of diluted deproteinized plasma was determined by its reduction to NO using vanadium chloride and hydrochloric acid and calibrated using sodium NO_3_^−^ standards.

#### 2.5.2. Mood

The Brunel Mood Scale (BRUMS) [50,51] is used to assess mood states in adult populations and was conducted prior to exercise as mood may have a mediating effect on resistance training performance [52]. Using the standard response timeframe of “How do you feel right now?”, 24 items representing six subscales (i.e., anger, confusion, depression, fatigue, tension, vigor; four items per subscale) were captured using a five-point Likert scale (i.e., 0 = not at all, 1 = a little, 2 = moderately, 3 = quite a bit, 4 = extremely). Respective items were summed so that each subscale score ranged from 0 to 16 raw points. In general, elevated vigor and decreased anger, confusion, depression, fatigue, and tension subscale scores are viewed as positive outcomes.

#### 2.5.3. Muscle Oxygenation

Throughout the entire duration of the experimental protocol on days 1 and 4, muscle oxygenation saturation status of the vastus lateralis and pectoralis major were monitored noninvasively via a portable, spatially resolved near-infrared spectroscopy device (Moxy Monitor, Fortiori Design LLC, Spicer, MN, USA), which has been previously tested for reliability and validity [53,54,55]. Briefly, the device uses four light-emitting diodes as light sources to continuously emit near-infrared light over the range 680–800 nm. The device features two light detectors that continuously monitor light intensity at each wavelength, with light source–detector separation distances of 12.5 and 25 mm. Data were collected at 0.5 Hz. Based upon the differing absorption spectra of oxygenated and deoxygenated hemoglobin + myoglobin within the near-infrared range, the device then used the modified Beer–Lambert Law to provide estimates of muscle O_2_ saturation (SmO_2_, as a % value) as: (oxygenated [hemoglobin + myoglobin]/total [hemoglobin + myoglobin]) × 100. This parameter is considered relatively insensitive to changes in blood volume and hence represents the balance between O_2_ delivery and utilization (and hence, O_2_ extraction) within the interrogated region [56]. The sensors were covered by a black rubber sheet during the test to minimize the intrusion of extraneous light and fixed along the belly of the vastus lateralis and pectoralis major using kinesiology tape and surgical tape. Marks were made around the sensor’s location using indelible ink during the first visit, and participants were asked to maintain these marks throughout the duration of the study to ensure precise placement for each subsequent visit. During the RTF protocol, baseline and end-exercise SmO_2_ were defined as the mean SmO_2_ values over the 20 s immediately prior to exercise onset and at the end of the exercise bout, respectively. The difference between baseline and end-exercise SmO_2_ (i.e., ΔSmO_2_) was calculated as an indicator of the amplitude of the response. Following exercise onset, SmO_2_ evidenced a time delay (TD) before systematically decreasing towards a nadir value; this TD was characterized as the first value following exercise onset, which differed from baseline SmO_2_ by more than 1 SD.

#### 2.5.4. Muscular Power, Velocity, and Endurance

Power and velocity measurements were obtained during exercise using a portable, wireless, commercially available, linear position transducer (GymAware, Kinetitech Performance Technology, Mitchell, Australia), which has been previously used [44] and validated for test–retest reliability [57]. During the 2 sets × 2 repetitions at 70%1RM, power and velocity were averaged across sets for the determination of mean power and mean velocity, and the highest power and velocity values were recorded for the determination of peak power and peak velocity. During the RTF set at 60%1RM, task failure was determined by the participant’s inability to complete the concentric phase of the lift. Peak power, mean power, peak velocity, mean velocity, and number of total repetitions completed for the single RTF set were recorded for analysis.

#### 2.5.5. Statistical Analyses 

Two-way (condition × time) repeated-measures ANOVAs were used to investigate statistical differences in plasma [NO_3_^−^] and [NO_2_^−^], mood, muscle oxygenation, and resistance exercise performance across time (day 1 vs. day 4) and between groups (PL and BR). Significant main and interaction effects were explored further using post hoc Bonferroni corrections. In addition, Student’s paired *t*-tests were used to determine physiological and performance differences where appropriate. Pearson product–moment correlation coefficients were used to assess the significant relationships between changes in plasma [NO_2_^−^] and performance variables. Unless stated otherwise, requisite statistical assumptions were met prior to all inferential analyses (e.g., sphericity, normality of the residuals, extreme outliers). Effect sizes for ANOVAs were measured via partial eta squared (*η_p_*^2^) in which small, medium, and large effects were operationalized as 0.01, 0.06, and 0.14, respectively [58]. Effect sizes for t-tests were measured as Cohen’s *d_z_* in which small, medium, and large effects were operationalized as 0.2, 0.5, and 0.8, respectively [58,59]. Statistical significance was set to *p* ≤ 0.05 with all data presented as mean ± SD, unless otherwise stated. All data were analyzed using SPSS version 26 (IBM, Armonk, NY, USA).

## 3. Results

All participants reported consuming all servings of each supplement at the correct times and confirmed that they had maintained their habitual exercise and dietary habits prior to each testing visit. There were no reports of adverse reactions, gastrointestinal distress or discomfort following ingestion of either supplement.

### 3.1. Plasma [NO_3_^–^] and [NO_2_^–^]

The within- and between-day CV% for plasma [NO_3_^–^] and [NO_2_^–^] were similar to previously reported values [60]. Specifically, for within-day duplicate samples, the CV% was ~8.9 ± 7.9% and ~3.0 ± 0.4% for plasma [NO_3_^–^] and [NO_2_^–^], respectively. The CV% for between day 1 and day 4 analyses was ~17.7 ± 20.0% and ~16.1 ± 2.0% for plasma [NO_3_^–^] and [NO_2_^–^], respectively. Plasma [NO_3_^–^] and [NO_2_^–^] are displayed in Table 1. There was an interaction effect (*p* < 0.001, *η_p_^2^* = 0.43), a main effect of condition (*p* < 0.001, *η_p_^2^* = 0.91), and a main effect of time (*p* < 0.05, *η_p_^2^* = 0.37) on plasma [NO_3_^–^]. Plasma [NO_3_^–^] was greater in BR compared to PL on day 1 (*p* < 0.001, *d_z_* = 2.91) and day 4 (*p* < 0.001, *d_z_* = 2.97) and was increased on day 4 compared to day 1 in BR (*p* = 0.01, *d_z_* = 0.79) but with no differences between days in PL (*p* > 0.05). There was a main effect of condition (*p* < 0.001, *η_p_^2^* = 0.66) but no effect of time (*p* > 0.05) or interaction effect (*p* > 0.05) on plasma [NO_2_^–^]. Compared to PL, plasma [NO_2_^–^] was higher in BR on day 1 (*p* < 0.001, *d_z_* = 1.23) and day 4 (*p* < 0.001, *d_z_* = 1.22) with no significant difference between days within conditions (*p* > 0.05). 

### 3.2. Mood

There was no effect of condition (*p* > 0.05) or time (*p* > 0.05) on the six subcategories of mood (Table 2).

### 3.3. Muscle Oxygenation

Owing to technical issues in one participant, analysis was completed in a subset of 13 out of 14 participants. There was no effect of condition (*p* > 0.05) or time (*p* > 0.05) on tissue saturation index in the vastus lateralis or pectoralis major during the explosive power protocols or RTF in the back squat and bench press (Table 3, Table 4, Table 5 and Table 6).

### 3.4. Muscular Power and Velocity 

Performance outcomes during the protocol for determining power and velocity during back squat and bench press are displayed in Table 7 and Table 8. There was no effect of condition (*p* > 0.05) in all performance variables during back squat and bench press. There was an effect of time (*p* < 0.05, *η_p_^2^* = 0.36) such that there was an increase from day 1 to day 4 in peak power in back squats for PL (*p* < 0.05, *d_z_* = 0.10), peak power in bench press for BR (*p* < 0.05, *d_z_* = 0.61), mean power in bench press for BR (*p* < 0.01, *d_z_* = 0.83), and mean velocity in bench press in BR (*p* < 0.01, *d_z_* = 1.01). 

### 3.5. Muscular Endurance Performance

**Figure 2:**
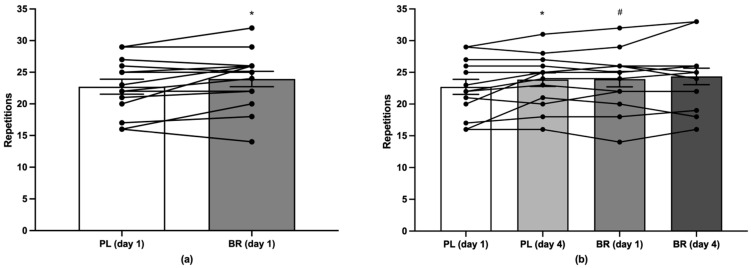
Data are means ± SE of the number of repetitions-to-failure (RTF) during bench press exercise (**a**) on day 1 between conditions and (**b**) on day 1 and 4 between conditions; RTF for individual participants shown in black lines. * = significantly different compared to day 1; # = significantly different compared to PL day 1; BR = NO_3_^−^-rich beetroot juice; PL = NO_3_^−^-depleted beetroot juice.

Performance outcomes during the protocol for determining muscular endurance during back squat and bench press are displayed in Table 9 and Table 10. There was a main effect of condition (*p* < 0.05, *η_p_*^2^ = 0.29) such that the number of RTF during bench press was increased in BR compared to PL by ~5% on day 1 (*p* < 0.05, *d_z_* = 0.55), with individual responses shown in Figure 2. There was a main effect of time (*p* < 0.01, *η_p_*^2^ = 0.44) on the number of RTF during bench press such that RTF increased from day 1 to day 4 in PL (*p* < 0.05, *d_z_* = 0.60). There was an effect of time on the number of RTF during back squat in BR (*p* < 0.05, *d_z_* = 0.62). 

## 4. Discussion

The main novel findings of the present study were that acute dietary NO_3_^−^ supplementation increased RTF during bench press (medium effect of *d_z_* = 0.55), but neither acute nor multiday NO_3_^−^ supplementation improved RTF during back squat, or muscular power, velocity, or oxygenation in either resistance exercise modality. In addition, compared to acute NO_3_^−^ ingestion, 4 days of NO_3_^−^ ingestion further elevated plasma [NO_3_^−^], but plasma [NO_2_^−^] was elevated by a similar magnitude after acute and multiday NO_3_^−^ supplementation. These findings are partially consistent with our hypotheses and suggest that dietary NO_3_^−^ supplementation is more efficacious for improving performance during upper-body, compared to lower-body, resistance exercise continued to failure. These results have implications for healthy active males seeking to improve resistance exercise performance.

### 4.1. Influence of Dietary Nitrate Supplementation on Resistance Exercise Performance

Acute NO_3_^−^ ingestion significantly increased the number of RTF during bench press by ~5% compared to PL. These results are in agreement with some [41,44] but not all [42] previous studies. The magnitude of improvement in bench press RTF in the current study was lower compared to previous studies reporting ~9% [44] and ~19% [41] improvements in RTF in free-weight and Smith-machine bench press, respectively. We also observed no effect of NO_3_^−^ supplementation on RTF for back squat, which is in agreement with one previous study [38], but contrasts with others [39,40,42]. Furthermore, we did not observe an impact of acute or multiday NO_3_^−^ supplementation on power output or velocity during back squat or bench press. To date, only four studies have examined the effect of NO_3_^−^ supplementation on power output during resistance exercise [40,42,43,44] with these studies yielding conflicting results. Indeed, NO_3_^−^ supplementation was ineffective at improving power output during back squat and bench press in one study [42], but most other studies have reported increased power during back squat (6% [40]; 18% [43]) and bench press (19% [44]) after NO_3_^−^ supplementation. Similar to the current study, these studies administered low (~6 mmol NO_3_^−^) to moderate (~12 mmol NO_3_^−^) NO_3_^−^ doses, which have been suggested to be more efficacious for improving muscular power compared to higher doses [30]. Therefore, the lack of an ergogenic effect of NO_3_^−^ supplementation on some resistance exercise performance variables in the current study is unlikely to be a result of an insufficient or too great a NO_3_^−^ dose. 

The principal novel finding of the current study was that an ergogenic effect of NO_3_^−^ supplementation was attained during bench press RTF, with no effect on this outcome variable during back squat exercise. This observation is compatible with the notion that dietary NO_3_^−^ supplementation may preferentially elicit beneficial physiological and functional effects on type II muscle fibers [22]. Specifically, since the upper-body musculature may comprise a greater proportion of type II muscle fibers compared to the lower-body musculature [46], this may have contributed to the observation herein that NO_3_^−^ supplementation resulted in exercise modality specific effects on RTF, which was improved after bench press but not back squat exercise. In addition, while there is evidence that dietary NO_3_^−^ supplementation can improve muscle power output during exercise commenced from an unfatigued state [11,21,28], there is also evidence to suggest that NO_3_^−^ supplementation may be more effective at enhancing neuromuscular function in a fatigued state [61]. Since the back squat RTF protocol always preceded bench press RTF protocol in the current study, this may have contributed to the ergogenic effect of NO_3_^−^ supplementation on bench press but not back squat RTF performance. This could also possibly explain why power and velocity of contraction was not impacted by NO_3_^−^ ingestion in the present study since the protocol for determining power was performed in an unfatigued state. However, it should be recognized that the reported effect of NO_3_^−^ supplementation exerting a greater effect on neuromuscular function in a fatigued compared to an unfatigued state was observed when local neuromuscular fatigue was elicited in the same muscle group (knee extensors) [61]. As such, the extent to which this transfers into the current study, with any changes in local fatigue in the upper-body musculature being achieved by prior fatigue in the back squat exercise, is unclear. Prior fatigue of a remote muscle group can expedite fatigue in another muscle group with such effects linked to greater central fatigue development [62]. However, dietary NO_3_^−^ supplementation does not appear to alter central fatigue development during exercise, at least during cycling exercise [63,64]. Further research is required to resolve the mechanisms for improved RTF during bench press exercise after NO_3_^−^ supplementation and whether such effects are more or less likely when preceded by prior fatiguing back squat exercise. It is also unclear whether NO_3_^−^ supplementation can improve performance during multiple sets of resistance exercise completed to failure, using the same exercise modality, where greater neuromuscular fatigue accumulates with successive fatiguing sets. A previous study reported that 7 days of dietary NO_3_^−^ supplementation preserved cycling sprint performance in the second of 2 × 40 min “halves” of a simulated team sport intermittent sprinting protocol, but not the first “half” [65] is also consistent with the assertion that NO_3_^−^ supplementation is more effective at enhancing performance as neuromuscular fatigue develops. Therefore, our findings are consistent with some previous observations that NO_3_^−^ supplementation has a greater effect on blunting neuromuscular fatigue development during fatiguing conditions or situations relying on a greater recruitment of type II fibers; however, further work is required to elucidate the impact of NO_3_^−^ supplementation when performance is assessed following fatigue development. The mechanisms that underlie this effect of NO_3_^−^ supplementation on muscle fatigue resistance remain unclear, but are likely to include improving calcium handling [15,16], lowered energy cost of force production [14], or the metabolic milieu during exercise [14]. 

### 4.2. Acute vs. Multiday NO_3_^−^ Supplementation 

The current study directly compared the effects of acute and multiday NO_3_^−^ supplementation, for the first time, on resistance exercise performance and found that only acute NO_3_^−^ supplementation enhanced RTF performance. These results suggest that acute NO_3_^−^ doses may be more effective at improving resistance exercise performance, at least for RTF. Our data support a recent meta-analysis that found there was a greater effect of NO_3_^−^ supplementation size on enhancing muscular power output following acute NO_3_^−^ ingestion compared to longer NO_3_^−^ supplementation periods [24]. In other exercise modalities, the few studies that include direct comparisons between acute and multiday NO_3_^−^ supplementation have reported conflicting results such that multiday NO_3_^−^ ingestion improved cycling performance [37,66], preserved the lowered O_2_ cost of exercise compared to acute NO_3_^−^ ingestion [67], but also had no effect on running performance [36]. Given the methodological discrepancies between existing studies that compared the efficacy of acute versus short-term NO_3_^−^ supplementation to improve performance, it is difficult to draw firm conclusions as to whether acute or multiday NO_3_^−^ supplementation is preferable for attaining performance enhancements during resistance exercise, which warrants further investigation. 

Acute NO_3_^−^ ingestion can rapidly elevate plasma [10] and muscle [7] NO_3_^−^, which provides substrate for additional NO production. Thus, acute NO_3_^−^ ingestion may elicit effects via second messenger cyclic guanosine monophosphate signaling to impact calcium sensitivity [68] or induce reversible post-translational modifications on key contractile proteins [69,70,71,72]. Since longer NO_3_^−^ supplementation periods could evoke structural changes to proteins that regulate contraction [1] and increase baseline levels of skeletal muscle and plasma NO_3_^−^, which would presumably lead to a greater capacity to maintain and/or elevate NO bioavailability on demand [9,73], we cannot exclude the possibility that extending the supplementation window beyond 4 days could have elicited an ergogenic effect, and further research is warranted. We observed that there was considerable interindividual variability in the magnitude of increase in plasma [NO_2_^−^] following NO_3_^−^ ingestion compared to placebo, where increases ranged from ~40% to ~570%; however, there was no correlation between the change in plasma [NO_2_^−^] and change in bench press RTF (*r* = −0.017), suggesting that a greater elevation to circulating [NO_2_^−^] did not increase the likelihood of ergogenic effects. It is also interesting to note that despite an additional increase in plasma [NO_3_^−^] with 4 days of NO_3_^−^ supplementation compared to 1 day, this did not translate into further elevations in plasma [NO_2_^−^] at day 4 versus acute supplementation. Other studies that implemented longer term NO_3_^−^ supplementation found that plasma [NO_3_^−^] increased over time [74] or stayed the same [67], whilst plasma [NO_2_^−^] remained at similar levels over time [37,67,74]. Additional research is required to elucidate the impact of acute and longer duration NO_3_^−^ supplementation periods on nitrate metabolism and performance effects during resistance exercise.

Power output in back squat and bench press, together with velocity in bench press, were increased on day 4 compared to day 1 in both conditions, and the reasons are unclear. However, possible explanations include a learning effect. Even though our study design included a randomization to the order and a familiarization session to abolish the learning effect, it could be possible that there was still a learning effect that occurred on neuromuscular performance during the four days of the first condition, which was subsequently removed during the washout period, but then re-emerged during the second condition [75,76]. 

### 4.3. Influence of NO_3_^−^ Supplementation on Muscle Oxygenation

In the present study, near-infrared spectroscopy was employed to provide insight into whether dietary NO_3_^−^ ingestion could impact the balance between local O_2_ delivery and muscle O_2_ utilization in skeletal muscle, specifically at the vastus lateralis and pectoralis major during back squat and bench press exercise, respectively. In contrast to our experimental hypothesis, there was no effect of acute or multiday NO_3_^−^ supplementation on tissue oxygenation during resistance exercise protocols for determining power, velocity, or endurance. To date, no other studies have examined the impact of NO_3_^−^ on tissue oxygenation during resistance exercise, but data reported in other exercise modalities yield conflicting results. For example, muscle oxygenation has been shown to improve during cycling exercise from transitions of low- to high-intensity work rates [17] and during faster cadences relative to slow cadences in cycling [20]. In contrast, NO_3_^−^ has also been shown to be ineffective at influencing muscle oxygenation [66,77,78,79]. In hypoxic states, NO_3_^−^ supplementation has been shown to preserve muscle oxygenation [80,81] but also shown to have no effect [82]. Therefore, our data suggest that improved bench press RTF after acute NO_3_^−^ supplementation is not linked to altered pectoralis major tissue oxygenation. However, we recognize that a continuous-wave NIRS device was used to assess tissue oxygenation in the current study and cannot exclude the possibility that NO_3_^−^ supplementation may have improved tissue oxygenation, which may have been detectable using a superior frequency-domain time-resolved NIRS system.

## 5. Conclusions

In conclusion, acute but not multiday NO_3_^−^-rich beetroot juice supplementation increased the number of repetitions completed prior to failure during bench press, but not during back squats, without influencing skeletal muscle oxygenation. Compared to acute NO_3_^−^ ingestion, four days of NO_3_^−^ supplementation increased plasma [NO_3_^−^] to a greater extent, but plasma [NO_2_^−^] was elevated similarly after acute and multiday NO_3_^−^-rich beetroot juice supplementation. There was no effect of NO_3_^−^ supplementation on power and velocity during the back squat or bench press exercise. These data provide original observations to improve understanding of the ergogenic potential of NO_3_^−^ supplementation to enhance resistance exercise performance and suggest that NO_3_^−^ supplementation may be effective at enhancing muscular endurance during upper-body exercise.

## Figures and Tables

**Table 1:** Plasma nitrate and nitrite concentrations following 1 and 4 days of dietary nitrate ingestion.

	PL		BR	
Variable	Day 1	Day 4	Day 1	Day 4
Plasma [NO_3_^−^] (µM)	39 ± 13	34 ± 13	428 ± 131 ^#^	506 ± 159 *^#^
Plasma [NO_2_^−^] (nM)	248 ± 82	236 ± 91	581 ± 272 ^#^	595 ± 286 ^#^

* = significantly different compared to day 1; ^#^ = significantly different between conditions; BR = NO_3_^−^-rich beetroot juice; NO_3_^−^ = nitrate; NO_2_^−^ = nitrite; PL = NO_3_^−^-depleted beetroot juice; nM = nanomolar; µM = micromolar.

**Table 2:** Summary of variations in mood across all experimental visits.

	PL				BR			
Variable	Day 1	Day 1 Mdn	Day 4	Day 4 Mdn	Day 1	Day 1 Mdn	Day 4	Day 4 Mdn
Anger	0.43 ± 0.85	0.00	0.29 ± 0.83	0.00	0.29 ± 0.61	0.00	0.21 ± 0.58	0.00
Confusion	0.07 ± 0.27	0.00	0.07 ± 0.27	0.00	0.43 ± 1.09	0.00	0.50 ± 1.09	0.00
Depression	0.29 ± 0.61	0.00	0.71 ± 1.98	0.00	0.43 ± 1.09	0.00	0.50 ± 0.94	0.00
Fatigue	3.43 ± 3.08	2.50	3.86 ± 3.78	3.00	3.07 ± 3.20	2.50	2.21 ± 2.29	1.50
Tension	0.57 ± 1.02	0.00	1.00 ± 1.92	0.00	1.00 ± 1.30	0.00	0.64 ± 0.84	0.00
Vigor	6.93 ± 3.20	8.00	6.36 ± 2.53	6.50	7.07 ± 2.64	6.50	6.64 ± 3.25	7.00

BR = NO_3_^−^ rich beetroot juice; Mdn = median; PL = NO_3_^−^ depleted beetroot juice.

**Table 3:** Muscle oxygenation of the vastus lateralis during a back squat exercise protocol for determination of power and velocity following acute and multiday nitrate supplementation.

	PL				BR			
	Day 1		Day 4		Day 1		Day 4	
Variable	Set 1	Set 2	Set 1	Set 2	Set 1	Set 2	Set 1	Set 2
Baseline (%)	71 ± 11	72 ± 11	71 ± 13	70 ± 12	71 ± 13	73 ± 10	72 ± 14	73 ± 13
TD (s)	4.2 ± 2.6	4.5 ± 2.5	4.6 ± 2.5	3.9 ± 3.3	2.6 ± 2.1	3.0 ± 3.3	4.9 ± 3.6	3.9 ± 3.4
Nadir during exercise	65 ± 16	68 ± 15	63 ± 17	61 ± 16	58 ± 17	63 ± 15	66 ± 15	65 ± 16
Nadir post-exercise	24 ± 17	26 ± 19	29 ± 25	22 ± 23	21 ± 16	20 ± 18	24 ± 17	25 ± 19
ΔSmO_2_ during exercise	−6 ± 11	−4 ± 8	−8 ± 9	−9 ± 10	−13 ± 14	−11 ± 15	−5 ± 8	−8 ± 10
ΔSmO_2_ total	−47 ± 13	−46 ± 14	−42 ± 20	−48 ± 17	−51 ± 15	−53 ± 15	−47 ± 17	−48 ± 17
Time to nadir post-exercise	22 ± 5	21 ± 6	22 ± 7	22 ± 6	20 ± 5	19 ± 5	23 ± 7	21 ± 6
Lag from end-exercise	16 ± 5	15 ± 6	14 ± 7	15 ± 6	13 ± 5	13 ± 5	16 ± 5	14 ± 4

Δ = difference; BR = NO_3_^−^-rich beetroot juice; PL = NO_3_^−^-depleted beetroot juice; s = seconds; SmO_2_ = muscle oxygen saturation; TD = time delay.

**Table 4:** Muscle oxygenation of the pectoralis major during a bench press exercise protocol for determination of power and velocity following acute and multiday nitrate supplementation.

	PL				BR			
	Day 1		Day 4		Day 1		Day 4	
Variable	Set 1	Set 2	Set 1	Set 2	Set 1	Set 2	Set 1	Set 2
Baseline (%)	82 ± 11	81 ± 12	80 ± 12	81 ± 11	80 ± 12	79 ± 15	77 ± 8	78 ± 9
TD (s)	2.6 ± 2.1	3.9 ± 2.1	2.9 ± 2.4	3.2 ± 2.3	4.2 ± 2.4	4.2 ± 2.7	3.1 ± 2.7	2.5 ± 2.7
Nadir during exercise	77 ± 16	78 ± 15	72 ± 16	76 ± 16	73 ± 15	78 ± 15	70 ± 11	70 ± 15
Nadir post-exercise	56 ± 24	60 ± 21	59 ± 20	61 ± 20	56 ± 25	58 ± 25	48 ± 26	50 ± 24
ΔSmO_2_ during exercise	−8 ± 12	−3 ± 5	−10 ± 12	−5 ± 9	−7 ± 7	−1 ± 8	−7 ± 8	−8 ± 11
ΔSmO_2_ total	−26 ± 17	−21 ± 13	−23 ± 17	−20 ± 16	−24 ± 18	−20 ± 17	−29 ± 22	−28 ± 18
Time to nadir post-exercise	14 ± 6	15 ± 6	14 ± 7	16 ± 9	16 ± 7	17 ± 8	17 ± 8	17 ± 8
Lag from end-exercise	9 ± 6	9 ± 7	9 ± 6	10 ± 9	11 ± 7	12 ± 8	11 ± 8	11 ± 8

Δ = difference; BR = NO_3_^−^-rich beetroot juice; PL = NO_3_^−^-depleted beetroot juice; s = seconds; SmO_2_ = muscle oxygen saturation; TD = time delay.

**Table 5:** Muscle oxygenation of the vastus lateralis during a repetitions-to-failure back squat exercise protocol for determination of muscular endurance following acute and multiday nitrate supplementation.

	PL		BR	
Variable	Day 1	Day 4	Day 1	Day 4
Baseline (%)	82 ± 10	82 ± 10	83 ± 11	85 ± 8
TD (s)	3.9 ± 6.0	2.6 ± 3.3	2.8 ± 3.5	3.2 ± 3.1
End-exercise SmO_2_ (%)	17 ± 18	13 ± 20	8 ± 6	10 ± 10
ΔSmO_2_ during exercise	−65 ± 21	−70 ± 19	−75 ± 15	−75 ± 11

Δ = difference; BR = NO_3_^−^-rich beetroot juice; PL = NO_3_^−^-depleted beetroot juice; s = seconds; SmO_2_ = muscle oxygen saturation; TD = time delay.

**Table 6:** Muscle oxygenation of the pectoralis major during a repetitions-to-failure bench press exercise protocol for determination of muscular endurance following acute and multiday nitrate supplementation.

	PL		BR	
Variable	Day 1	Day 4	Day 1	Day 4
Baseline (%)	84 ± 9	86 ± 8	84 ± 12	82 ± 9
TD (s)	9.9 ± 10.7	13.4 ± 14.6	8.9 ± 12.0	9.5 ± 12.0
End-exercise SmO_2_ (%)	68 ± 27	61 ± 28	56 ± 28	54 ± 23
ΔSmO_2_ during exercise	−15 ± 22	−25 ± 26	−27 ± 22	−28 ± 20

Δ = difference; BR = NO_3_^−^-rich beetroot juice; PL = NO_3_^−^-depleted beetroot juice; s = seconds; SmO_2_ = muscle oxygen saturation; TD = time delay.

**Table 7:** Performance outcomes during a back squat exercise protocol for determination of power and velocity following acute and multiday nitrate supplementation.

	PL		BR	
Variable	Day 1	Day 4	Day 1	Day 4
Peak Power (W)	1700 ± 444	1810 ± 478 *	1736 ± 461	1778 ± 461
Mean Power (W)	699 ± 163	693 ± 189	689 ± 162	707 ± 162
Peak Velocity (m/s)	1.5 ± 0.3	1.5 ± 0.2	1.5 ± 0.2	1.5 ± 0.1
Mean Velocity (m/s)	0.7 ± 0.1	0.7 ± 0.1	0.8 ± 0.1	0.8 ± 0.1

* = significantly different compared to day 1; BR = NO_3_^−^-rich beetroot juice; PL = NO_3_^−^-depleted beetroot juice; m/s = meters per second; W = watts.

**Table 8:** Performance outcomes during a bench press exercise protocol for determination of power and velocity following acute and multiday nitrate supplementation.

	PL		BR	
Variable	Day 1	Day 4	Day 1	Day 4
Peak Power (W)	665 ± 179	725 ± 216	659 ± 163	693 ± 183 *
Mean Power (W)	411 ± 107	438 ± 124	404 ± 90	431 ± 114 *
Peak Velocity (m/s)	0.9 ± 0.2	0.9 ± 0.1	0.9 ± 0.2	0.9 ± 0.2
Mean Velocity (m/s)	0.6 ± 0.1	0.6 ± 0.1	0.6 ± 0.1	0.6 ± 0.1 *

* = significantly different compared to day 1; BR = NO_3_^−^-rich beetroot juice; PL = NO_3_^−^-depleted beetroot juice; m/s = meters per second; W = watts.

**Table 9:** Performance outcomes during a back squat exercise protocol for determination of muscular endurance following acute and multiday nitrate supplementation.

	PL		BR	
Variable	Day 1	Day 4	Day 1	Day 4
Repetitions	28 ± 9	29 ± 10	28 ± 7	30 ± 7 *
Peak Power (W)	843 ± 229	897 ± 255	869 ± 235	845 ± 204
Mean Power (W)	493 ± 150	502 ± 166	497 ± 152	486 ± 131
Peak Velocity (m/s)	0.9 ± 0.1	1.0 ± 0.1	0.9 ± 0.1	0.9 ± 0.1
Mean Velocity (m/s)	0.6 ± 0.1	0.6 ± 0.1	0.6 ± 0.1	0.6 ± 0.1

* = significantly different compared to day 1; BR = NO_3_^−^-rich beetroot juice; PL = NO_3_^−^-depleted beetroot juice; m/s = meters per second; W = watts.

**Table 10:** Performance outcomes during a bench press exercise protocol for determination of muscular endurance following acute and multiday nitrate supplementation.

	PL		BR	
Variable	Day 1	Day 4	Day 1	Day 4
Reps	23 ± 4	24 ± 4 *	24 ± 5 ^#^	24 ± 5
Peak Power (W)	461 ± 186	508 ± 273	466 ± 241	468 ± 192
Mean Power (W)	309 ± 88	333 ± 135	309 ± 118	308 ± 90
Peak Velocity (m/s)	0.8 ± 0.1	0.7 ± 0.1	0.7 ± 0.1	0.7 ± 0.1
Mean Velocity (m/s)	0.5 ± 0.1	0.5 ± 0.1	0.5 ± 0.1	0.5 ± 0.1

* = significantly different compared to day 1; ^#^ = significantly different compared to PL day 1; BR = NO_3_^−^-rich beetroot juice; PL = NO_3_^−^-depleted beetroot juice; m/s = meters per second; W = watts.

## Data Availability

Not applicable.

## References

[1] Stamler J.S., Meissner G. (2001). Physiology of Nitric Oxide in Skeletal Muscle. Physiol. Rev..

[2] Lundberg J.O., Weitzberg E., Gladwin M.T. (2008). The Nitrate-Nitrite-Nitric Oxide Pathway in Physiology and Therapeutics. Nat. Rev. Drug Discov..

[3] Hezel M.P., Weitzberg E. (2015). The Oral Microbiome and Nitric Oxide Homoeostasis. Oral Dis..

[4] Shiva S., Huang Z., Grubina R., Sun J., Ringwood L.A., MacArthur P.H., Xu X., Murphy E., Darley-Usmar V.M., Gladwin M.T. (2007). Deoxymyoglobin Is a Nitrite Reductase That Generates Nitric Oxide and Regulates Mitochondrial Respiration. Circ. Res..

[5] Castello P.R., David P.S., McClure T., Crook Z., Poyton R.O. (2006). Mitochondrial Cytochrome Oxidase Produces Nitric Oxide under Hypoxic Conditions: Implications for Oxygen Sensing and Hypoxic Signaling in Eukaryotes. Cell Metab..

[6] Modin A., Björne H., Herulf M., Alving K., Weitzberg E., Lundberg J.O. (2001). Nitrite-Derived Nitric Oxide: A Possible Mediator of “acidic-Metabolic” Vasodilation. Acta Physiol. Scand..

[7] Kadach S., Piknova B., Black M.I., Park J.W., Wylie L.J., Stoyanov Z., Thomas S.M., McMahon N.F., Vanhatalo A., Schechter A.N. (2022). Time Course of Human Skeletal Muscle Nitrate and Nitrite Concentration Changes Following Dietary Nitrate Ingestion. Nitric Oxide.

[8] Nyakayiru J., Kouw I.W.K., Cermak N.M., Senden J.M., van Loon L.J.C., Verdijk L.B. (2017). Sodium Nitrate Ingestion Increases Skeletal Muscle Nitrate Content in Humans. J. Appl. Physiol..

[9] Wylie L.J., Park J.W., Vanhatalo A., Kadach S., Black M.I., Stoyanov Z., Schechter A.N., Jones A.M., Piknova B. (2019). Human Skeletal Muscle Nitrate Store: Influence of Dietary Nitrate Supplementation and Exercise. J. Physiol..

[10] Wylie L.J., Kelly J., Bailey S.J., Blackwell J.R., Skiba P.F., Winyard P.G., Jeukendrup A.E., Vanhatalo A., Jones A.M. (2013). Beetroot Juice and Exercise: Pharmacodynamic and Dose-Response Relationships. J. Appl. Physiol..

[11] Coggan A.R., Broadstreet S.R., Mikhalkova D., Bole I., Leibowitz J.L., Kadkhodayan A., Park S., Thomas D.P., Thies D., Peterson L.R. (2018). Dietary Nitrate-Induced Increases in Human Muscle Power: High versus Low Responders. Physiol. Rep..

[12] Porcelli S., Ramaglia M., Bellistri G., Pavei G., Pugliese L., Montorsi M., Rasica L., Marzorati M. (2015). Aerobic Fitness Affects the Exercise Performance Responses to Nitrate Supplementation. Med. Sci. Sports Exerc..

[13] Wilkerson D.P., Hayward G.M., Bailey S.J., Vanhatalo A., Blackwell J.R., Jones A.M. (2012). Influence of Acute Dietary Nitrate Supplementation on 50 Mile Time Trial Performance in Well-Trained Cyclists. Eur. J. Appl. Physiol..

[14] Bailey S.J., Fulford J., Vanhatalo A., Winyard P.G., Blackwell J.R., DiMenna F.J., Wilkerson D.P., Benjamin N., Jones A.M. (2010). Dietary Nitrate Supplementation Enhances Muscle Contractile Efficiency during Knee-Extensor Exercise in Humans. J. Appl. Physiol..

[15] Bailey S.J., Gandra P.G., Jones A.M., Hogan M.C., Nogueira L. (2019). Incubation with Sodium Nitrite Attenuates Fatigue Development in Intact Single Mouse Fibres at Physiological PO_2_. J. Physiol..

[16] Hernández A., Schiffer T.A., Ivarsson N., Cheng A.J., Bruton J.D., Lundberg J.O., Weitzberg E., Westerblad H. (2012). Dietary Nitrate Increases Tetanic [Ca2+]i and Contractile Force in Mouse Fast-Twitch Muscle. J. Physiol..

[17] Breese B.C., McNarry M.A., Marwood S., Blackwell J.R., Bailey S.J., Jones A.M. (2013). Beetroot Juice Supplementation Speeds O2 Uptake Kinetics and Improves Exercise Tolerance during Severe-Intensity Exercise Initiated from an Elevated Metabolic Rate. Am. J. Physiol. Regul. Integr. Comp. Physiol..

[18] Ferguson S.K., Hirai D.M., Copp S.W., Holdsworth C.T., Allen J.D., Jones A.M., Musch T.I., Poole D.C. (2013). Impact of Dietary Nitrate Supplementation via Beetroot Juice on Exercising Muscle Vascular Control in Rats. J. Physiol..

[19] Petrick H.L., Brownell S., Vachon B., Brunetta H.S., Handy R.M., van Loon L.J.C., Murrant C.L., Holloway G.P. (2022). Dietary Nitrate Increases Submaximal SERCA Activity and ADP-Transfer to Mitochondria in Slow-Twitch Muscle of Female Mice. Am. J. Physiol. Endocrinol. Metab..

[20] Bailey S.J., Varnham R.L., DiMenna F.J., Breese B.C., Wylie L.J., Jones A.M. (2015). Inorganic Nitrate Supplementation Improves Muscle Oxygenation, O₂ Uptake Kinetics, and Exercise Tolerance at High but Not Low Pedal Rates. J. Appl. Physiol..

[21] Coggan A.R., Leibowitz J.L., Kadkhodayan A., Thomas D.P., Ramamurthy S., Spearie C.A., Waller S., Farmer M., Peterson L.R. (2015). Effect of Acute Dietary Nitrate Intake on Maximal Knee Extensor Speed and Power in Healthy Men and Women. Nitric Oxide.

[22] Jones A.M., Ferguson S.K., Bailey S.J., Vanhatalo A., Poole D.C. (2016). Fiber Type-Specific Effects of Dietary Nitrate. Exerc. Sport Sci. Rev..

[23] Andersen L.L., Andersen J.L., Zebis M.K., Aagaard P. (2010). Early and Late Rate of Force Development: Differential Adaptive Responses to Resistance Training?. Scand. J. Med. Sci. Sports.

[24] Coggan A.R., Baranauskas M.N., Hinrichs R.J., Liu Z., Carter S.J. (2021). Effect of Dietary Nitrate on Human Muscle Power: A Systematic Review and Individual Participant Data Meta-Analysis. J. Int. Soc. Sports Nutr..

[25] Jonvik K.L., Nyakayiru J., Van Dijk J.W., Maase K., Ballak S.B., Senden J.M.G., Van Loon L.J.C., Verdijk L.B. (2018). Repeated-Sprint Performance and Plasma Responses Following Beetroot Juice Supplementation Do Not Differ between Recreational, Competitive and Elite Sprint Athletes. Eur. J. Sport Sci..

[26] Jonvik K.L., Hoogervorst D., Peelen H.B., de Niet M., Verdijk L.B., van Loon L.J.C., van Dijk J.-W. (2021). The Impact of Beetroot Juice Supplementation on Muscular Endurance, Maximal Strength and Countermovement Jump Performance. Eur. J. Sport Sci..

[27] Kokkinoplitis K., Chester N. (2014). The Effect of Beetroot Juice on Repeated Sprint Performance and Muscle Force Production. JPES.

[28] Wylie L.J., Bailey S.J., Kelly J., Blackwell J.R., Vanhatalo A., Jones A.M. (2016). Influence of Beetroot Juice Supplementation on Intermittent Exercise Performance. Eur. J. Appl. Physiol..

[29] Coggan A.R., Hoffman R.L., Gray D.A., Moorthi R.N., Thomas D.P., Leibowitz J.L., Thies D., Peterson L.R. (2020). A Single Dose of Dietary Nitrate Increases Maximal Knee Extensor Angular Velocity and Power in Healthy Older Men and Women. J. Gerontol. A Biol. Sci. Med. Sci..

[30] Gallardo E.J., Gray D.A., Hoffman R.L., Yates B.A., Moorthi R.N., Coggan A.R. (2021). Dose—Response Effect of Dietary Nitrate on Muscle Contractility and Blood Pressure in Older Subjects: A Pilot Study. J. Gerontol. Ser. A.

[31] Cuenca E., Jodra P., Pérez-López A., González-Rodríguez L.G., Fernandes da Silva S., Veiga-Herreros P., Domínguez R. (2018). Effects of Beetroot Juice Supplementation on Performance and Fatigue in a 30-s All-Out Sprint Exercise: A Randomized, Double-Blind Cross-Over Study. Nutrients.

[32] Domínguez R., Garnacho-Castaño M.V., Cuenca E., García-Fernández P., Muñoz-González A., de Jesús F., Lozano-Estevan M.D.C., Fernandes da Silva S., Veiga-Herreros P., Maté-Muñoz J.L. (2017). Effects of Beetroot Juice Supplementation on a 30-s High-Intensity Inertial Cycle Ergometer Test. Nutrients.

[33] Jodra P., Domínguez R., Sánchez-Oliver A.J., Veiga-Herreros P., Bailey S.J. (2020). Effect of Beetroot Juice Supplementation on Mood, Perceived Exertion, and Performance During a 30-Second Wingate Test. Int. J. Sports Physiol. Perform..

[34] Kramer S.J., Baur D.A., Spicer M.T., Vukovich M.D., Ormsbee M.J. (2016). The Effect of Six Days of Dietary Nitrate Supplementation on Performance in Trained CrossFit Athletes. J. Int. Soc. Sports Nutr..

[35] Rimer E.G., Peterson L.R., Coggan A.R., Martin J.C. (2016). Increase in Maximal Cycling Power With Acute Dietary Nitrate Supplementation. Int. J. Sports Physiol. Perform..

[36] Boorsma R.K., Whitfield J., Spriet L.L. (2014). Beetroot Juice Supplementation Does Not Improve Performance of Elite 1500-m Runners. Med. Sci. Sports Exerc..

[37] Vanhatalo A., Bailey S.J., Blackwell J.R., DiMenna F.J., Pavey T.G., Wilkerson D.P., Benjamin N., Winyard P.G., Jones A.M. (2010). Acute and Chronic Effects of Dietary Nitrate Supplementation on Blood Pressure and the Physiological Responses to Moderate-Intensity and Incremental Exercise. Am. J. Physiol. Regul. Integr. Comp. Physiol..

[38] Flanagan S.D., Looney D.P., Miller M.J.S., DuPont W.H., Pryor L., Creighton B.C., Sterczala A.J., Szivak T.K., Hooper D.R., Maresh C.M. (2016). The Effects of Nitrate-Rich Supplementation on Neuromuscular Efficiency during Heavy Resistance Exercise. J. Am. Coll Nutr..

[39] Garnacho-Castaño M.V., Sánchez-Nuño S., Molina-Raya L., Carbonell T., Maté-Muñoz J.L., Pleguezuelos-Cobo E., Serra-Payá N. (2022). Circulating Nitrate-Nitrite Reduces Oxygen Uptake for Improving Resistance Exercise Performance after Rest Time in Well-Trained CrossFit Athletes. Sci. Rep..

[40] Jurado-Castro J.M., Campos-Perez J., Ranchal-Sanchez A., Durán-López N., Domínguez R. (2022). Acute Effects of Beetroot Juice Supplements on Lower-Body Strength in Female Athletes: Double-Blind Crossover Randomized Trial. Sports Health.

[41] Mosher S.L., Sparks S.A., Williams E.L., Bentley D.J., Mc Naughton L.R. (2016). Ingestion of a Nitric Oxide Enhancing Supplement Improves Resistance Exercise Performance. J. Strength Cond Res..

[42] Ranchal-Sanchez A., Diaz-Bernier V.M., De La Florida-Villagran C.A., Llorente-Cantarero F.J., Campos-Perez J., Jurado-Castro J.M. (2020). Acute Effects of Beetroot Juice Supplements on Resistance Training: A Randomized Double-Blind Crossover. Nutrients.

[43] Rodríguez-Fernández A., Castillo D., Raya-González J., Domínguez R., Bailey S.J. (2021). Beetroot Juice Supplementation Increases Concentric and Eccentric Muscle Power Output. Original Investigation. J. Sci. Med. Sport.

[44] Williams T.D., Martin M.P., Mintz J.A., Rogers R.R., Ballmann C.G. (2020). Effect of Acute Beetroot Juice Supplementation on Bench Press Power, Velocity, and Repetition Volume. J. Strength Cond. Res..

[45] Polgar J., Johnson M.A., Weightman D., Appleton D. (1973). Data on Fibre Size in Thirty-Six Human Muscles. An Autopsy Study. J. Neurol. Sci..

[46] Zinner C., Morales-Alamo D., Ørtenblad N., Larsen F.J., Schiffer T.A., Willis S.J., Gelabert-Rebato M., Perez-Valera M., Boushel R., Calbet J.A.L. (2016). The Physiological Mechanisms of Performance Enhancement with Sprint Interval Training Differ between the Upper and Lower Extremities in Humans. Front. Physiol..

[47] Baranauskas M.N., Freemas J.A., Tan R., Carter S.J. (2022). Moving beyond Inclusion: Methodological Considerations for the Menstrual Cycle and Menopause in Research Evaluating Effects of Dietary Nitrate on Vascular Function. Nitric Oxide.

[48] Govoni M., Jansson E.A., Weitzberg E., Lundberg J.O. (2008). The Increase in Plasma Nitrite after a Dietary Nitrate Load Is Markedly Attenuated by an Antibacterial Mouthwash. Nitric Oxide.

[49] Wilk M., Golas A., Stastny P., Nawrocka M., Krzysztofik M., Zajac A. (2018). Does Tempo of Resistance Exercise Impact Training Volume?. J. Hum. Kinet..

[50] Terry P.C., Lane A.M., Lane H.J., Keohane L. (1999). Development and Validation of a Mood Measure for Adolescents. J. Sports Sci..

[51] Terry P.C., Lane A.M., Fogarty G.J. (2003). Construct Validity of the Profile of Mood States-Adolescents for Use with Adults. Psychol. Sport Exerc..

[52] Beedie C.J., Terry P.C., Lane A.M. (2000). The Profile of Mood States and Athletic Performance: Two Meta-Analyses. J. Appl. Sport Psychol..

[53] Crum E.M., O’Connor W.J., Van Loo L., Valckx M., Stannard S.R. (2017). Validity and Reliability of the Moxy Oxygen Monitor during Incremental Cycling Exercise. Eur. J. Sport Sci..

[54] Feldmann A., Schmitz R.W., Erlacher D. (2019). Near-Infrared Spectroscopy-Derived Muscle Oxygen Saturation on a 0% to 100% Scale: Reliability and Validity of the Moxy Monitor. J. Biomed. Opt..

[55] McManus C.J., Collison J., Cooper C.E. (2018). Performance Comparison of the MOXY and PortaMon Near-Infrared Spectroscopy Muscle Oximeters at Rest and during Exercise. J. Biomed. Opt..

[56] Ferrari M., Muthalib M., Quaresima V. (2011). The Use of Near-Infrared Spectroscopy in Understanding Skeletal Muscle Physiology: Recent Developments. Philos. Trans. A Math. Phys. Eng. Sci..

[57] Ballmann C.G., McCullum M.J., Rogers R.R., Marshall M.R., Williams T.D. (2021). Effects of Preferred vs. Nonpreferred Music on Resistance Exercise Performance. J. Strength Cond. Res..

[58] Cohen J. (1988). Statistical Power Analysis for the Behavioral Sciences.

[59] Lakens D. (2013). Calculating and Reporting Effect Sizes to Facilitate Cumulative Science: A Practical Primer for *t*-Tests and ANOVAs. Front. Psychol..

[60] Tan R., Wylie L.J., Wilkerson D.P., Vanhatalo A., Jones A.M. (2022). Effects of Dietary Nitrate on the O2 Cost of Submaximal Exercise: Accounting for “Noise” in Pulmonary Gas Exchange Measurements. J. Sports Sci..

[61] Tillin N.A., Moudy S., Nourse K.M., Tyler C.J. (2018). Nitrate Supplement Benefits Contractile Forces in Fatigued but Not Unfatigued Muscle. Med. Sci. Sports Exerc..

[62] Johnson M.A., Sharpe G.R., Williams N.C., Hannah R. (2015). Locomotor Muscle Fatigue Is Not Critically Regulated after Prior Upper Body Exercise. J. Appl. Physiol..

[63] Husmann F., Bruhn S., Mittlmeier T., Zschorlich V., Behrens M. (2019). Dietary Nitrate Supplementation Improves Exercise Tolerance by Reducing Muscle Fatigue and Perceptual Responses. Front. Physiol..

[64] Thurston T.S., Weavil J.C., Hureau T.J., Gifford J.R., Georgescu V.P., Wan H.-Y., La Salle D.T., Richardson R.S., Amann M. (2021). On the Implication of Dietary Nitrate Supplementation for the Hemodynamic and Fatigue Response to Cycling Exercise. J. Appl. Physiol..

[65] Thompson C., Wylie L.J., Fulford J., Kelly J., Black M.I., McDonagh S.T.J., Jeukendrup A.E., Vanhatalo A., Jones A.M. (2015). Dietary Nitrate Improves Sprint Performance and Cognitive Function during Prolonged Intermittent Exercise. Eur. J. Appl. Physiol..

[66] Jo E., Fischer M., Auslander A.T., Beigarten A., Daggy B., Hansen K., Kessler L., Osmond A., Wang H., Wes R. (2019). The Effects of Multi-Day vs. Single Pre-Exercise Nitrate Supplement Dosing on Simulated Cycling Time Trial Performance and Skeletal Muscle Oxygenation. J. Strength Cond. Res..

[67] Wylie L.J., Ortiz de Zevallos J., Isidore T., Nyman L., Vanhatalo A., Bailey S.J., Jones A.M. (2016). Dose-Dependent Effects of Dietary Nitrate on the Oxygen Cost of Moderate-Intensity Exercise: Acute vs. Chronic Supplementation. Nitric Oxide.

[68] Coggan A.R., Peterson L.R. (2018). Dietary Nitrate Enhances the Contractile Properties of Human Skeletal Muscle. Exerc. Sport Sci. Rev..

[69] Dutka T.L., Mollica J.P., Lamboley C.R., Weerakkody V.C., Greening D.W., Posterino G.S., Murphy R.M., Lamb G.D. (2017). S-Nitrosylation and S-Glutathionylation of Cys134 on Troponin I Have Opposing Competitive Actions on Ca^2+^ Sensitivity in Rat Fast-Twitch Muscle Fibers. Am. J. Physiol. Cell Physiol..

[70] Gould N., Doulias P.-T., Tenopoulou M., Raju K., Ischiropoulos H. (2013). Regulation of Protein Function and Signaling by Reversible Cysteine S-Nitrosylation. J. Biol. Chem..

[71] Ishii T., Sunami O., Saitoh N., Nishio H., Takeuchi T., Hata F. (1998). Inhibition of Skeletal Muscle Sarcoplasmic Reticulum Ca ^2+^ -ATPase by Nitric Oxide. FEBS Lett..

[72] Nogueira L., Figueiredo-Freitas C., Casimiro-Lopes G., Magdesian M.H., Assreuy J., Sorenson M.M. (2009). Myosin Is Reversibly Inhibited by S-Nitrosylation. Biochem. J..

[73] Nyakayiru J., van Loon L.J.C., Verdijk L.B. (2020). Could Intramuscular Storage of Dietary Nitrate Contribute to Its Ergogenic Effect? A Mini-Review. Free Radic. Biol. Med..

[74] Whitfield J., Ludzki A., Heigenhauser G.J.F., Senden J.M.G., Verdijk L.B., van Loon L.J.C., Spriet L.L., Holloway G.P. (2016). Beetroot Juice Supplementation Reduces Whole Body Oxygen Consumption but Does Not Improve Indices of Mitochondrial Efficiency in Human Skeletal Muscle. J. Physiol..

[75] Mattocks K.T., Buckner S.L., Jessee M.B., Dankel S.J., Mouser J.G., Loenneke J.P. (2017). Practicing the Test Produces Strength Equivalent to Higher Volume Training. Med. Sci. Sports Exerc..

[76] Nuzzo J.L., Taylor J.L., Gandevia S.C. (2019). CORP: Measurement of Upper and Lower Limb Muscle Strength and Voluntary Activation. J. Appl Physiol..

[77] Kent G.L., Dawson B., Cox G.R., Abbiss C.R., Smith K.J., Croft K.D., Lim Z.X., Eastwood A., Burke L.M., Peeling P. (2018). Effect of Dietary Nitrate Supplementation on Thermoregulatory and Cardiovascular Responses to Submaximal Cycling in the Heat. Eur. J. Appl. Physiol..

[78] Nybäck L., Glännerud C., Larsson G., Weitzberg E., Shannon O.M., McGawley K. (2017). Physiological and Performance Effects of Nitrate Supplementation during Roller-Skiing in Normoxia and Normobaric Hypoxia. Nitric Oxide.

[79] Rokkedal-Lausch T., Franch J., Poulsen M.K., Thomsen L.P., Weitzberg E., Kamavuako E.N., Karbing D.S., Larsen R.G. (2021). Multiple-Day High-Dose Beetroot Juice Supplementation Does Not Improve Pulmonary or Muscle Deoxygenation Kinetics of Well-Trained Cyclists in Normoxia and Hypoxia. Nitric Oxide.

[80] Masschelein E., Van Thienen R., Wang X., Van Schepdael A., Thomis M., Hespel P. (2012). Dietary Nitrate Improves Muscle but Not Cerebral Oxygenation Status during Exercise in Hypoxia. J. Appl. Physiol..

[81] Shannon O.M., Duckworth L., Barlow M.J., Deighton K., Matu J., Williams E.L., Woods D., Xie L., Stephan B.C.M., Siervo M. (2017). Effects of Dietary Nitrate Supplementation on Physiological Responses, Cognitive Function, and Exercise Performance at Moderate and Very-High Simulated Altitude. Front. Physiol..

[82] Cocksedge S.P., Breese B.C., Morgan P.T., Nogueira L., Thompson C., Wylie L.J., Jones A.M., Bailey S.J. (2020). Influence of Muscle Oxygenation and Nitrate-Rich Beetroot Juice Supplementation on O_2_ Uptake Kinetics and Exercise Tolerance. Nitric Oxide.

